# The What, the When, and the Whether of Intentional Action in the Brain: A Meta-Analytical Review

**DOI:** 10.3389/fnhum.2017.00238

**Published:** 2017-05-17

**Authors:** Laura Zapparoli, Silvia Seghezzi, Eraldo Paulesu

**Affiliations:** ^1^fMRI Unit, IRCCS Istituto Ortopedico GaleazziMilan, Italy; ^2^Psychology Department and NeuroMI—Milan Centre for Neuroscience, University of Milano-BicoccaMilan, Italy

**Keywords:** intentional action, motor control, fMRI, PET, meta-analysis

## Abstract

In their attempt to define discrete subcomponents of intentionality, Brass and Haggard (2008) proposed their *What, When, and Whether model* (*www*-*model*) which postulates that the content, the timing and the possibility of generating an action can be partially independent both at the cognitive level and at the level of their neural implementation. The original proposal was based on a limited number of studies, which were reviewed with a discursive approach. To assess whether the model stands in front of the more recently published data, we performed a systematic review of the literature with a meta-analytic method based on a hierarchical clustering (HC) algorithm. We identified 15 PET/fMRI studies well-suited for this quest. HC revealed the existence of a rostro-caudal gradient within the medial prefrontal cortex, with the more anterior regions (the anterior cingulum) involved in more abstract decisions of whether to execute an action and the more posterior ones (the middle cingulum or the SMA) recruited in specifying the content and the timing components of actions. However, in contrast with the original *www*-model, this dissociation involves also brain regions well outside the median wall of the frontal lobe, in a component specific manner: the supramarginal gyrus for the *what* component, the pallidum and the thalamus for the *when* component, the putamen and the insula for the *whether* component. We then calculated co-activation maps on the three component-specific *www* clusters of the medial wall of the frontal/limbic lobe: to this end, we used the activation likelihood approach that we applied on the imaging studies on action contained in the BrainMap.org database. This analysis confirmed the main findings of the HC analyses. However, the BrainMap.org data analyses also showed that the aforementioned segregations are generated by paradigms in which subjects act in response to conditional stimuli rather than while driven by their own intentions. We conclude that the available data confirm that the neural underpinnings of intentionality can be fractionated in discrete components that are partially independent. We also suggest that intentionality manifests itself in discrete components through the boosting of general purpose action-related regions specialized for different aspects of action selection and inhibition.

## Introduction

Motor control has been the object of interest of many disciplines, including psychology, cognitive neuroscience, and, since the earliest days, philosophy, particularly when the object of enquiry are the conscious aspects of motor control and intentionality (Jeannerod, 1997; Frith et al., 2000; Haggard, 2008).

A comprehensive model that differentiates the various stages of movement production is not available: even articulated models of motor control (see for example Frith et al., 2000) remain underspecified, especially when considering the intentional components and their neural implementation.

In an earlier attempt to provide a model of the brain bases of intentionality, Jahanshahi (1998) argued that, in principle, it should be possible to characterize intentional actions in at least three main components: the content (*what* component), the timing (*when* component) and the possibility of being executed or inhibited (*whether* component). However, in reviewing the then available neuroimaging literature, they concluded that there was no sufficient evidence that such components are represented in discrete brain circuits. In their unitary brain model of intentionality, they proposed a “system” for intentional actions located in the pre-frontal cortex, anterior cingulate cortex, and supplementary motor area (SMA), with subcortical inputs coming from the striatum and through the thalamus: in their views, these systems are responsible for the all three features of intentional action (Jahanshahi, 1998).

Ten years later, Brass and Haggard (2008) re-assessed the early insights of Jahanshahi (1998) and, taking advantage of a larger set of imaging data, proposed that a “What, When, and Whether” model (*www-model*)^1^ of intentional action is justified also on anatomical grounds. The original *www-model* was based on a set of experiments that we review here briefly together with more recent observations.

### “*What*” component

The *What* component has been mostly investigated by using fMRI procedures similar to the “Free selection paradigm” (Lau et al., 2004b), in which two experimental conditions are compared: a condition in which responses are externally determined by a cue and a condition in which the participants have to choose freely between different motor responses. Typically, the *What* component has been related with the activation of the fronto-medial cortex at the level of the rostral cingulate zone (Deiber et al., 1991; Frith et al., 1991; Hyder et al., 1997; Lau et al., 2004a,b; Mueller et al., 2007; Krieghoff et al., 2009), the SMA (Lau H. C. et al., 2006; van Eimeren et al., 2006) and pre-SMA (Deiber et al., 1991; Lau et al., 2004a,b; Brass and Haggard, 2007).

### “*When*” component

The timing component of intentional actions has been investigated by using the paradigm of Libet (Libet et al., 1983). For example, Lau et al. (2004a) associated the judgment of the onset of the intention to move with activation of the pre-SMA (Lau et al., 2004a). Libet' s paradigm has a number of limitations (see for example Trevena and Miller, 2002; Lau H. et al., 2006; Miller et al., 2011), first and foremost, of being meta-cognitive in nature and perhaps not terribly well-suited to fMRI given the temporal resolution of the technique and the time scale of the neurophysiological events seen with EEG during the paradigm.

The *When* component has been explored also by Jahanshahi et al. (1995) and Jenkins et al. (2000) who compared self-initiated extensions of the index finger with fingers' extensions triggered by pacing tones at unpredictable intervals: they found an activation of the dorsolateral prefrontal cortex specifically for the self-paced condition (Jahanshahi et al., 1995; Jenkins et al., 2000).

Finally, in an early and solitary attempt to dissociate the anatomical bases of the *What* and the *When* components in the same experiment, Hoffstaedter et al. (2013) manipulated the content and the timing of the motor responses of their participants. They found activations of the SMA, the insula, the globus pallidus, and the anterior putamen in relation to the free selection of the action's timing and the activation of the pre-SMA and the dorsal premotor cortex in relation to the free selection of the actions' content (Hoffstaedter et al., 2013).

### “*Whether*” component

The absence of a motor response as the result of the choice of action inhibition has partly hindered the study of the intentional inhibition processes with an explicit experimental task. The Libet' s task has been the main paradigm used to investigate voluntary inhibition. Using fMRI, Brass and Haggard have shown that an area of the dorsal and rostral fronto-medial cortex is more active when participants intentionally inhibit a response rather than when they complete the same action (Brass and Haggard, 2007). In any event, the voluntary inhibition of actions has been recently studied also with novel tasks like in the case of the *marble task* (Kühn et al., 2009; Schel et al., 2014) or the motivation driven *pain avoidance paradigm* of Lynn et al. (2016). These experiments showed that intentional inhibition rely on a neural network that includes parietal and lateral prefrontal cortex bilaterally (Kühn et al., 2009; Schel et al., 2014) and the pre-SMA (Schel et al., 2014; Lynn et al., 2016).

### Aims of the study

After the initial proposal of the *www-model*, some new ground has been covered with new explicit experiments to justify a formal assessment of the model, this time with explicit meta-analytical techniques.

Is the segregation of different components of the *www-model* justified by the new evidence? If so, does it involve specific portions of the medial wall of the frontal lobe and of the cingulate gyrus? Further, does the mapping of the discrete components involve other brain regions in a specific manner? Again, if so, is it possible, with all the needed caution, to infer from these additional regions on the nature of the subcomponents of intentionality postulated by the original model?

Further, are the regions involved in intentionality anatomically specific or do they simply contribute to this aspect of behavior while being also involved in conditional aspects of action selection?

These were all lingering questions on the *www-model* that we tried to address in the present study. To this end, we first used hierarchical clustering (HC) to identify component specific clusters. As the reader will see, the specific literature available is barely sufficient to make statistical inferences on the significance of the clusters identified. However, after the initial hierarchical clustering procedure, we interrogated the vast BrainMap.org database and generated co-activation maps based on the main component-specific medial wall clusters of the frontal lobe. This permitted the desired statistical assessment of the anatomical dissociations initially identified by hierarchical clustering.

## Materials and methods

### Data collection and preparation

Our meta-analysis was based on neuroimaging articles investigating the neurofunctional correlates of intentional action using PET or fMRI in adult subjects.

Candidate studies were selected through the PubMed database (http://www.ncbi.nlm.nih.gov/pubmed/). The search keys were: “Intentional action & fMRI” and “Intentional action & PET.” These queries returned 27 neuroimaging articles investigating the neurofunctional correlates of intentional actions. We included only studies that did satisfy the following inclusion criteria: (1) sample population composed of normal adult subjects; (2) imaging technique: PET or fMRI; (3) data reported using stereotactic coordinates; (4) comparison between intentional actions and stimulus-driven actions. As a consequence of these inclusion criteria, 12 studies were excluded from the analysis. Among the 15 studies that satisfied our inclusion criteria, 6 studies investigated the *What* component, 3 studies the *When* component, and 4 studies the *Whether* component. Finally, 2 studies investigated both the *What* and the *When* components. These studies were then classified on the basis of the examined component (see Supplementary Table 1).

For the suitable studies, in the meta-analysis we used data derived from (i) within group *simple effects* and (ii) interaction effects. The simple effects were: “Intention driven trials” and “Stimulus driven trials.” The interaction effects^2^ were “Intention driven trials > Stimulus driven trials” and vice versa. As there were no sufficient local maxima for the contrasts designed to identify the stimulus driven acts, these were not analyzed any further.

To summarize, the data selection led us to analyse 150 stereotactic activation loci, *71* peaks associated with the *What* component, *42* peaks with the When component and *37* peaks with the Whether component of intentional actions.

### Classification of the raw data prior to clustering analyses

For each activation peak, we recorded all relevant information about the statistical comparison that generated it, the nature of the experimental task and the investigated component.

We therefore determined a list of classification criteria to characterize each peak of activation included in the dataset:

– t-values or z-scores;
– Sample size;
– Average age of the subjects;
– Stereotactic template (MNI or Tailarach and Tournoux template);
– Whole brain or region-of-interest analysis;
– Scanning Technique (PET or fMRI);
– Statistical thresholds and nature of the correction for multiple comparisons.

In order to combine the data coming from studies based on different stereotactic spaces, the stereotactic coordinates of studies in which activation peaks were reported in terms of the Talairach and Tournoux (1988) atlas were transformed into the MNI (Montreal Neurological Institute) stereotactic space using Matthew Brett's procedure as implemented in the software GingerAle (www.brainmap.org).

### Clustering procedure

We first performed a component specific hierarchical clustering analyses (HC) of the activation peaks: the analysis allowed us to extract the principal clusters of regional effects from the database (Cattinelli et al., 2013) for each www component.

Hierarchical clustering was performed by using functions implemented in MATLAB 2016a. After computation of squared Euclidean distances between each pair of the input data, clusters with minimal dissimilarity were recursively merged using Ward's (1963) criterion which minimizes total intra-cluster variance after each merging step. As described in Cattinelli et al. (2013) and Crepaldi et al. (2013), “*this procedure results in a tree, whose leaves represent singletons (i.e., clusters formed of a single activation peak), and whose root represents one large cluster including all the activation peaks submitted to the algorithm. Each level of the tree reports the clusters created by the algorithm at a specific processing step, as it progresses from individual activation peaks at the lowest level to the all-inclusive final cluster at the top of the tree.”* The procedure was continued until the average standard deviation around the cluster centroids of the individual peaks, in the *x, y*, and *z* directions, remained below 5.0 mm. This measure roughly mimics the spatial resolution of fMRI studies. As hierarchical clustering may be sensitive to the order in which the individual data are processed, and may generate alternative clustering trees when integers are used (Morgan and Ray, 1995), an optimal clustering solution was identified by accepting the solution with maximized the between cluster error sum of squares (see for example Cattinelli et al., 2013). The mean coordinates of each cluster included in the final set were then passed as an input to a MATLAB script to automatically label the anatomical correspondence of the stereotactic coordinates of the centroids of each cluster. This procedure implied a query of the Automatic Anatomical Labeling (AAL) template available in the MRIcron visualization Software (Rorden and Brett, 2000). The initial automatic anatomical assignations were then double-checked by the authors with direct visual inspection on the AAL template and, if needed, the corresponding volumetric MRI scan template Ch2Bet released with the MRIcron software.

Given the number of peaks available for each analysis, relevant clusters at this stage were identified on the basis of the numerosity of the peaks in each cluster. Clusters were further considered if they contained a number of coordinates greater than the median of the distribution for each analysis and, in any event, with no <3 coordinates.

### Activation likelihood analyses

To assess to what extent the functional segregations identified by the HC analyses could be replicated on a much larger dataset of studies on action, we interrogated the BrainMap.org database to generate co-activation maps for the *What, When*, and *Whether* patterns using the Activation Likelihood Estimate approach (Turkeltaub et al., 2002; Eickhoff et al., 2012). Co-activation maps are essentially estimates of the probability that local effects, expressed as triplets of stereotactic coordinates, co-occur in a data-set together with the activation of a seed reference region. The advantage of the ALE approach is that the statistical significance of the clusters identified is assessed using formal statistical thresholding. In this case, three separate co-activation maps were calculated using as seeds the three specific clusters identified in the medial wall of the frontal/limbic lobe, as they were also postulated in the original www-model. The interrogation of the BrainMap.org database was performed by using a 10 mm wide 3D region of interest defined around the centroid of each cluster (the coordinates are indicated in bold font in Table 1).

**Table 1:** **Hierarchical clustering analysis**.

**Brain regions (BA)**	**MNI coordinates**									
	**Left hemisphere**					**Right hemisphere**				
	**Cluster ID**	***x***	***y***	***z***	***K***	**Cluster ID**	***x***	***y***	***z***	***K***
**A. WHAT COMPONENT**										
Frontal_Inf_Tri (46)^*^	12	−35	33	27	6					
Frontal_Mid (46)^*^						8	36	32	34	9
**Cingulum_Mid (24)**						**4**	**4**	**20**	**38**	**10**
SupraMarginal (40)^*^						18	47	−42	45	6
**B. WHEN COMPONENT**										
Frontal_Inf_Oper^*^	13	−45	13	5	3					
Rolandic_Oper^*^						11	45	7	10	3
Frontal_Mid (46)^*^						16	35	39	31	4
**Supp_Motor_Area (6)**						**9**	**5**	−**3**	**62**	**4**
Parietal_Inf (40)						6	46	−43	47	5
Angular^*^						5	34	−51	37	3
Pallidum^*^	12	−20	5	−2	3	10	21	5	1	3
**C. WHETHER COMPONENT**										
Frontal_Inf_Orb (47)	15	−40	32	−4	5					
Cingulum_Ant (11)						16	12	37	2	3
**Cingulum_Mid (24)**	**17**	−**1**	**29**	**35**	**3**					
Insula^*^						13	40	21	0	5
Thalamus	4	−1	−20	7	3					
Putamen	14	−26	5	−6	3					

In BOLD the stereotactic coordinates used as centroids for subsequent the co-activation maps analyses using the Activation Likelihood Estimate technique.

* *Regions shown also by the co-activation maps analyses for each centroid. K = number of peaks in the cluster*.

We interrogated all the studies that in the database are classified with the key-word “*action*” and “*activation*.”

The aim of these analyses was three-fold: (1) on the one hand we wanted to test the hypothesis that co-activation maps generated starting from the three main clusters of the medial wall of the frontal lobe and of the cingulate cortex can produce similarly segregated results as in the specific hierarchical clustering, once the three maps were formally compared (2) further, we wanted to test the hypothesis on whether the brain regions outside the frontal lobe would co-vary with the original seeds in a similar manner (3) by analyzing the composition of the BrainMap.org data-base experiments, that contributed to the identification of medial-wall clusters, we wanted to learn to what extent these could be associated, even if loosely, to intentional action and its subcomponents at the center of our quest.

As an initial step, three separate co-activation maps were calculated for each of the three seed clusters identified by the HC procedure. Subsequently, each co-activation map was compared with the other two lumped together.

Furthermore, once a component specific cluster was identified in the BrainMap.org database, we explored its composition as far as the generative paradigms was concerned. We paid particular attention to the relative contribution of go/no-go paradigms as these are closer to aspects of motor control entailed by the intentional/conditional dichotomy, particularly for the whether component.

## Results

### Hierarchical clustering

**Figure 1:**
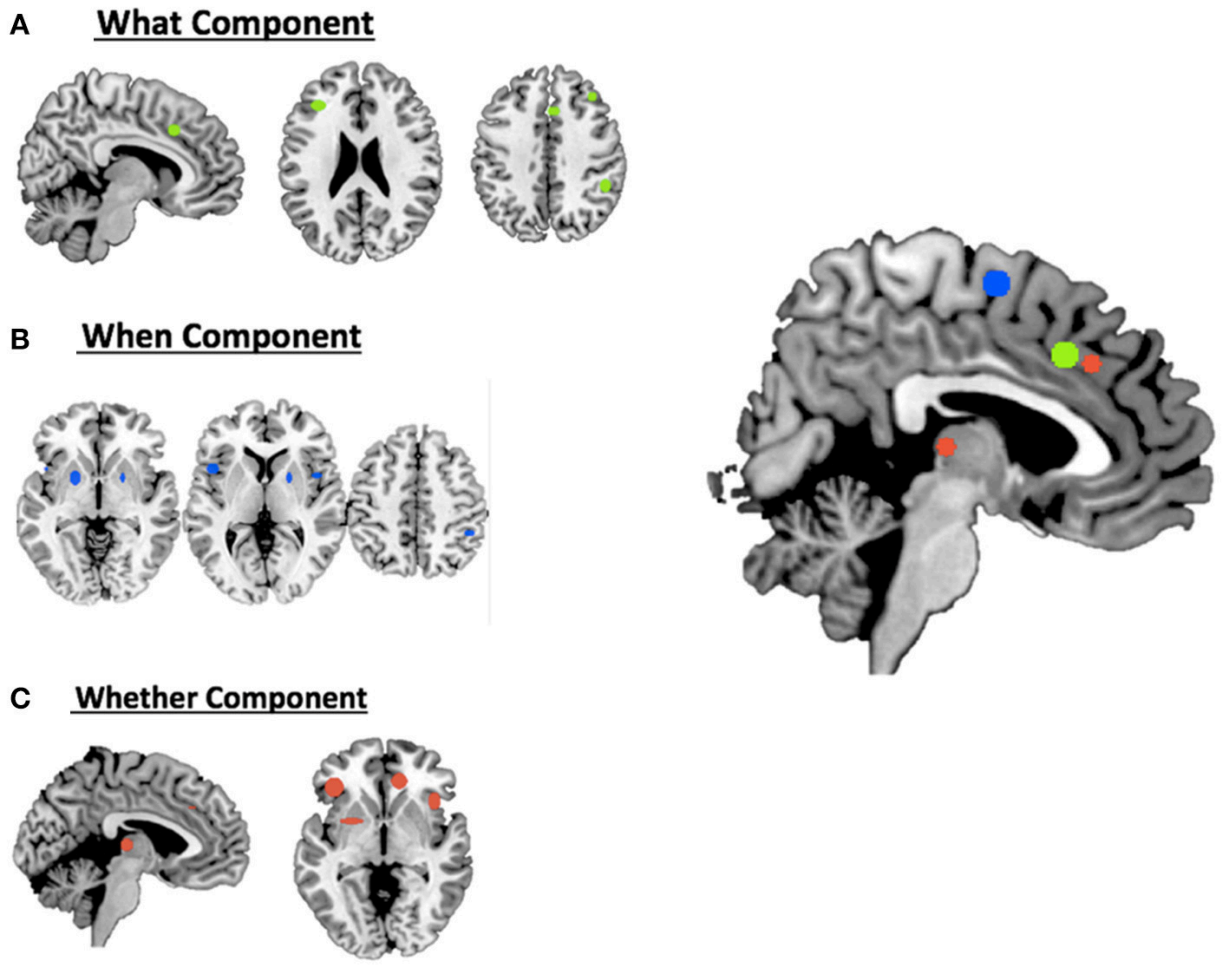
**Hierarchical clustering analysis results for (A)** the what component; **(B)** the when component; **(C)** the whether component and a summary of all the components on the medial view of the brain.

For the ***what*** component the data clustered in the right middle cingulum, the right frontal middle gyrus, the right supramarginal gyrus and the left inferior triangular frontal gyrus (see Table 1A and Figure 1A).

For the ***when*** component we found clusters in the right SMA, the right frontal middle gyrus, in the right inferior parietal lobule, in the right rolandic operculum, in the left inferior opercular frontal gyrus and in the lenticular nucleus bilaterally (see Table 1B and Figure 1B).

Finally, for the ***whether*** component, specific clusters were seen at the level of the right anterior and the left middle cingulum, the left inferior orbital frontal gyrus, the right insula and subcortical structures, such as the thalamus and the putamen (see Table 1C and Figure 1C).

### Co-activation maps

The BrainMap.org search for co-activations on the *What, When*, and *Whether* clusters of the medial frontal lobe wall (based on the centroids in bold in Table 1) retrieved 1,201 foci from 56 statistical comparisons for the *What* cluster, 1,488 foci from 73 statistical comparisons for the *When* cluster, and 614 foci from 24 statistical comparisons for the *Whether* component.

**Figure 2:**
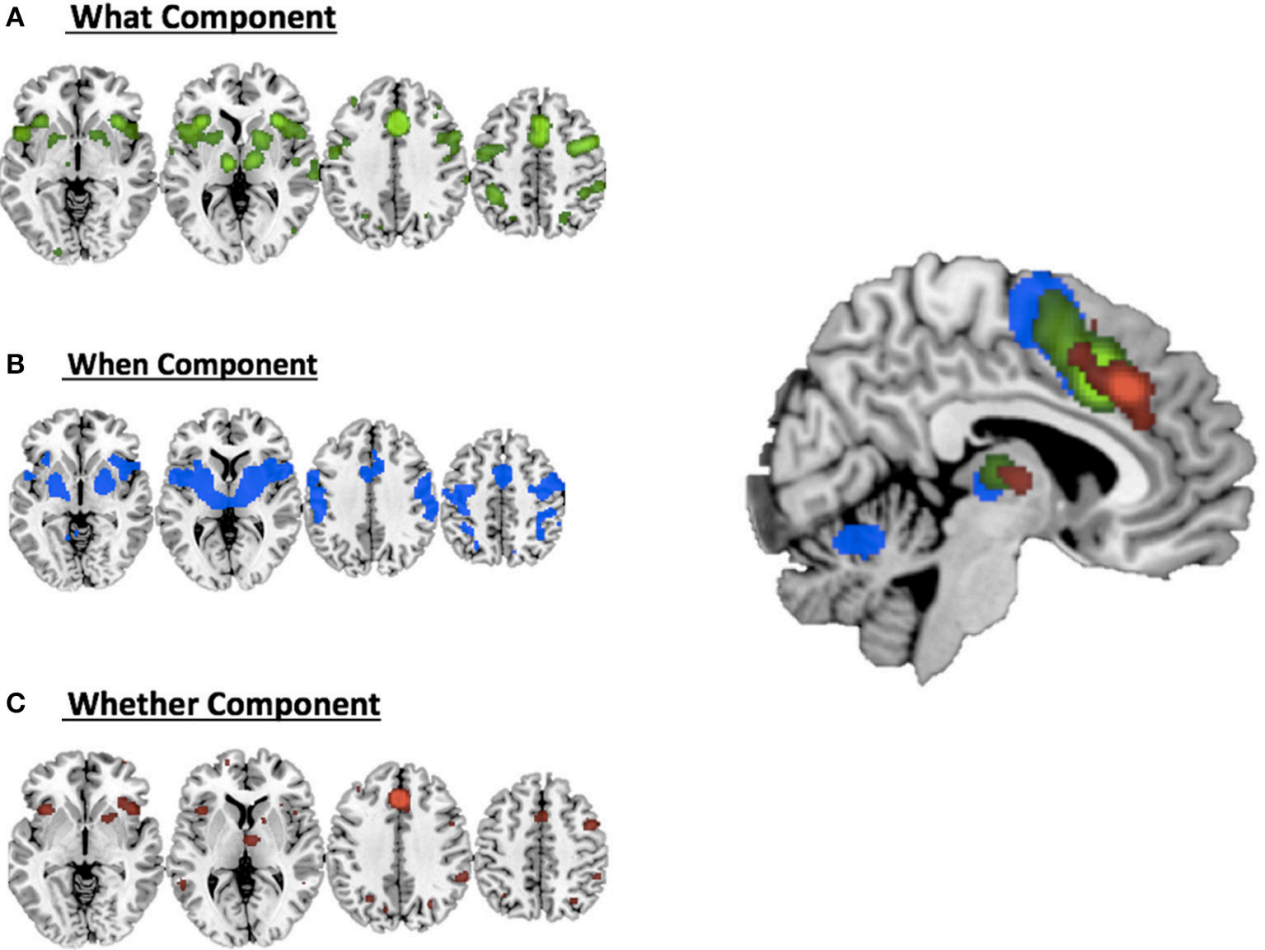
**Co-activations maps calculated with ALE for (A)** the what component; **(B)** the when component; **(C)** the whether component and a summary of all the components on the medial view of the brain.

Figure 2 illustrates the co-activation maps calculated around the original clusters used as seeds for the interrogation of the BrainMap.org database (statistical threshold *p* < 0.05 FDR corrected).

It should be noted that when seen as simple effects (e.g., the co-activation map generated by the ***what*** seed alone) most of the regions implicated by the HC analysis were also identified by the co-activation maps analysis (regions indicated with the symbol ^*^ in Table 1). Their relative segregation and embedding is illustrated in Figure 2.

Each individual map was then compared with a combination of the other two maps: for example, the ***What*** map vs. ***When*** & ***Whether*** ones combined, and so forth. For these comparisons, a more lenient *p* < 0.005 uncorrected threshold (cluster size threshold 300 mm^3^) was used.

These analyses led to identification of regions that genuinely dissociate on the basis of their co-activation with the seeds used.

**Figure 3:**
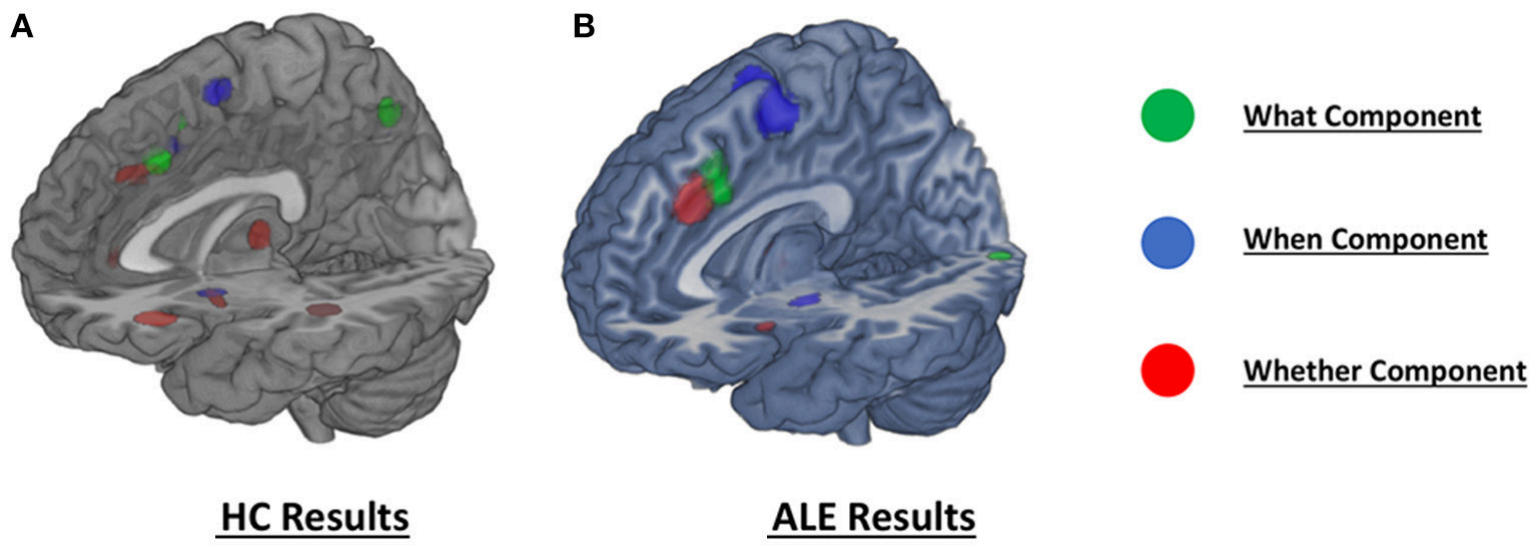
**Comparison of the dissociation between components assessed with (A)** hierarchical clustering analysis results and **(B)** ALE co-activation maps.

As illustrated in Figure 3B and summarized in Tables 2A–C, the comparative co-activation maps analysis confirms the functional dissociation along the medial wall of the frontal lobe, in a caudo-rostral direction, into a *what, when, and whether* components. Together with these brain regions, there were co-segregations of component-specific regions, particularly for the lenticular nuclei for the *when* component (see Figures 3A,B for a comparison between the two analyses).

**Table 2:** **Co-activation maps analyses**.

**Brain regions (BA)**	**MNI coordinates**									
	**Left hemisphere**					**Right hemisphere**				
	**Cluster ID**	**mm^3^**	***x***	***y***	***z***	**Cluster ID**	**mm^3^**	***x***	***y***	***z***
**A. WHAT COMPONENT**										
Frontal_Inf_Tri	2	856	−39.3	14	27.7					
Frontal_Inf_Oper (44)	2	856	−42	10	30					
Frontal_Mid_R (6)						3	416	32	4	50
Cingulum_Mid_R (24)						1	4760	3.7	18.5	40.3
Precentral_R (6)						3	416	34	−2	42
Insula_R						5	304	36	24	4
Temporal_Pole_Sup_L	6	200	−48	12	−2					
Calcarine_L (18)	4	304	−17	−97	−4					
**B. WHEN COMPONENT**										
Frontal_Inf_Oper (44)	7	816	−56	7	16					
			−62	8	18					
Frontal_Mid (8)	9	232	−32	−10	58					
Frontal_Sup (6)						4	7432	32	−10	66
Supp_Motor_Area						1	7432	4.2	−4.2	61.8
Precentral_gyrus (6)						8	424	63	6	18
Precentral gyrus (4)	13	56	−40	−24	62	4	7432	38	−16	58
						4	2952	55.5	−21.5	32.9
Postcentral gyrus (3)	11	168	−43	−36	61	4	2952	58	−16	38
	6	920	−42	−22.7	42.7					
			−42	−24	38					
Precuneus						5	1232	16.2	−50.8	−25.6
Cerebellum_6	12	136	−34	−62	−20	4	7432	40	−10	54
Cerebellum_Crus1	10	216	−22	−66	−30					
Putamen						2	4008	31.7	−1.2	7.1
Pallidum	3	3216	−23.6	−10.4	1.9	2	2952	22	−4	0
Thalamus	3	3216	−10	−26	10					
**C. WHETHER COMPONENT**										
Rolandic_Oper (44)	10	80	−46	24	18					
Cingulum_Mid (24)	1	3368	−1	29.2	34.6					
Precentral (6)						6	128	44	8	46
Insula	3	288	−36	14	−2	5	136	44	20	−8
Parietal_Inf	9	96	−34	−60	44					
Temporal_Mid (21)	2	456	−60	−48	4					
	7	120	−56	−54	6					
Putamen						4	136	20	12	−8
Thalamus L/R	2	456	0	−9	4	2	456	0	−9	4
Caudate	8	120	−10	6	18					
		120	−14	6	18					

*A, What > (When and Whether) from seed at x = 4; y = 20; z = 38*.

*B, When > (What and Whether) from seed at x = 5; y = −3; z = 62*.

*C, Whether > (What and When) from seed at x = −1; y = 29; z = 35*.

We also assessed whether the foci that contributed to the segregation of the component specific clusters of the median wall of the frontal lobe were associated with specific tasks. Tables 3A–C describes the nature of the tasks behind the regional effects. First, it should be noted that these did not contain foci from the studies that were entered in the present meta-analysis. Second, after an initial qualitative scrutiny, we compared the relative prevalence of go/no-go paradigms vs. other kind of paradigms.

**Table 3:** **Composition analysis of the medial frontal wall clusters isolated by the comparative co-activation maps analyses performed on the BrainMap.org dataset**.

	**Go/No-Go**	**Other**	**Chi Squared value**	***P*-value**
**A**				
**What**	17	39		
vs.			10.6	0.001
**When**	6	67		
**B**				
**Whether**	9	14		
vs.			12.86	0.0004
**When**	6	67		
**C**				
**Whether**	9	14		
vs.			0.57	0.451
**What**	17	39		

*The table reports the comparison of the proportion of regional effects derived from go/no-go paradigms and all other paradigms within each of the three clusters from the medial wall of the frontal/limbic lobe*.

*A, Chi-Squared Test—What vs. When*.

*B, Chi-Squared Test—Whether vs. When*.

*C, Chi-Squared Test—Whether vs. What*.

We then performed three Chi-squared pairwise comparisons between component specific cluster compositions to find that the relative prevalence of go/no-go data was significantly greater for the **whether** and **what** clusters in comparison with the data that generated the **when** cluster (see Table 3).

## Discussion

Contrary to previous anatomically “unitary” models of willed action (Jahanshahi, 1998), Brass and Haggard (2008) proposed the existence of a distributed meso-frontal system, responsible for discrete aspects of intentionality, encoding what to do, when to do it or whether or not to do it. However, in both the cited models, the authors based their conclusions on a qualitative analysis of the available PET/fMRI literature, characterized by a number of different experimental approaches.

The current meta-analytic study was conducted in order to further test these alternative neurocognitive models of intentional action, by formally exploring, as quantitatively as possible, the available literature. The results of our combination of hierarchical clustering and ALE meta-analytical procedures expands the initial model of Brass and Haggard (2008) showing that (1) a segregation of intention specific regions is possible even though the regions involved go beyond the mesial wall of the frontal lobe; (2) the regions involved coincide with brain areas that are active also for conditional (non-intentional) motor behaviors. This latter finding suggests that intentionality manifests itself in discrete components through the boosting of general purpose action-related regions specialized for different aspects of action selection and inhibition.

### A multi-component neural model of intentionality

Our results partially confirm the assumption of Brass and Haggard's model (2008), suggesting the existence of a rostro-caudal gradient within the medial prefrontal cortex, with the more anterior regions involved in more abstract decisions of whether to execute an action and the more posterior ones recruited in specifying the content and, yet more dorsally, the timing components of actions.

For example, for the decision about which action to perform (*what* component) the data clustered at the level of the right middle cingulum; the middle cingulum has been previously associated to conflict processing and conflict monitoring (Botvinick et al., 2004; Carter and van Veen, 2007) and it could contribute to the specific resolution of an internal conflict about which action to execute among different alternatives.

For what concerns the decision about the timing of the action, we found a cluster in the right SMA; this region has been previously explicitly linked to the timing or to the intentional initiation of a movement (Cunnington et al., 2003; Debaere et al., 2003). The association between the SMA and timing of intentional action is supported by recent studies on Parkinson's disease (PD). PD is characterized by difficulties in implementing intentional behaviors, but this impairment is reduced in the presence of an external salient cue, such as, for example, a fire (Jahanshahi, 1998); for this reason, it has been widely hypothesized that PD patients have a malfunctioning of the internal timing of action (Brass and Haggard, 2008). In PD patients, the SMA has abnormal connectivity with the thalamus, especially during the OFF-medication phase (Michely et al., 2015).

Finally, the studies investigating the intentional decision about whether or not to act converged in a cluster at the level of the right cingulum in its anterior portion, a region more anterior than the ones involved in the other two components. This finding confirms that the intentional inhibition of an action involves separate neural structures fleshing out the concept that deciding whether to act or not is separable from other aspects of intentionality.

### Going beyond the frontal mesial cortex

In contrast with the original www-model, the dissociations for the different components involved also brain regions well outside the median wall of the frontal lobe, in a component specific manner.

For example, for the *what* component the data clustered at the level of the right supramarginal gyrus, a finding confirmed by the co-activation maps analysis. The involvement of the parietal lobule in the decision about which action to execute should not be a surprise: the inferior parietal lobules are in fact critical nodes for the representation of actions and intentions to act according to previous findings (Tunik et al., 2007). Recently, Gallivan et al. (2011) showed that intentions for specific movements could be predicted by the spatial activity patterns in these areas. Moreover, direct electrical stimulation of the right inferior parietal lobule induces a strong intention to move; at relatively high stimulation intensities (~8 mA) patients may even feel an illusory sense of movement (Desmurget et al., 2009). Thus, in the context of intentional action, the parietal lobule seems to contribute to movement intention and motor awareness with specific reference to specific body parts.

For what concerns the internal timing of the action, a specific cluster was found in the frontal operculum, a structure previously associated with the synchronization of voluntary hand movements to an auditory rhythm (Thaut, 2003). It is also telling the involvement of the motor component of the lenticular nuclei bilaterally: these are of course part of a cortico-subcortical-cortical network that regulates motor behavior (Graybiel, 1998) and that is dysfunctional in movement disorders (see review in Crittenden and Graybiel, 2011).

Finally, for the *whether* component, our data clustered also at the level of the right anterior insula; anterior insula activations have been reported in various studies on response inhibition (Wager et al., 2005). Moreover, there is evidence indicating that anterior insula is associated with concentration and “cognitive effort” (Allen et al., 2007): if so, its involvement in this class of tasks, may represent the strain to decide whether to do or not to do something after this has already been planned.

We found two more clusters associated with the whether component, at the level of the thalamus and the putamen; the thalamus, and the basal ganglia in general, are known to play a crucial role in action selection (Humphries and Gurney, 2002; Humphries et al., 2006). The specific involvement of such structures in the intentional inhibition of actions is supported by their abnormal functioning in Gilles de la Tourette Syndrome, a movement disorder characterized by the presence of unwanted movements that patients are not usually able to inhibit (see review in Zapparoli et al., 2015): this neurological disease is most likely associated with aberrant activity in the basal ganglia and with functional changes in the cortico-striato-thalamo-cortical (CSTC) circuits (see Mink, 2006; Leckman et al., 2010; Felling and Singer, 2011; Ganos et al., 2013). A malfunction of the same circuitry has been described also in the obsessive-compulsive disorder (OCD, see for example Bandelow et al., 2016). The spectrum of OCD symptoms is too broad to be readily accommodated by malfunctions of the www-neural circuitry alone. However, as much as complex motor tics can be very similar to motor compulsions (Worbe et al., 2010), OCD symptoms are frequently observed in patients with Gilles de la Tourette Syndrome. Considerations about their frequent comorbidity suggest that the co-occurrence of the two syndromes may in fact represent a specific clinical entity, the recently defined Obsessive-Compulsive Tic Disorder (OCTD; see Dell'Osso et al., 2017): this may comprise the “malfunctioning” of the neural systems associated with the *whether* component of intentionality, explaining the difficulty of these patients to inhibit their compulsive/ticking behaviors.

### The functional neural correlates of intentional action and of action in general

The issue of whether the anatomy of intentional action, and its subcomponents, involves brain regions generally responsible for action was assessed by the co-activation maps analyses. This analysis confirmed the segregation of the clusters along the medial wall and the additional regions seen for the *www* components by our hierarchical clustering analysis. It also confirmed by far and large the extension of component specific regions outside the medial wall of the frontal/limbic lobe.

A composition analysis of the paradigms that contributed to each of the three clusters of the medial wall of the frontal/limbic lobe^3^ revealed, as one could expect, first and foremost the extreme heterogeneity of the paradigms that were retrieved with the only constraint of the key-words “action” and “activation” and “normal subjects” (see the Supplementary Table 2). Having identified the relative proportion of go/no-go paradigms behind each cluster, the *what* and the *whether* ones proved to have a significant larger proportion of such paradigms. Although some aspects of these findings are open to discussion, it is a matter of little surprise to observe that the *whether* component of intentionality maps into a cluster that contains a fair proportion of go/no-go paradigms in a general database of experiments on action.

However, the analysis of the paradigms that contributed the raw data for co-activation maps were far from being associated with intentional action experiments only. In fact, at the time of this writing (February 2017), as strange as it might seem^4^, the BrainMap.org database did not contain the 15 studies that were submitted to our hierarchical clustering analysis on the studies on intention. This fortuitous feature was instrumental to our analyses as we were guaranteed that the hierarchical clustering and the co-activation maps analyses were independent, adding validity to our inferences.

In returning to the crucial matter of our contention here, the fact that the medial wall seeds, on which the co-activation maps analyses were based, segregate in an identical manner to what is revealed by the hierarchical clustering performed on “intentional” experiments, suggests that the specificity of intentionality and its subcomponents cannot be sought *phrenologically* in terms of minute segregated regions exclusively involved in intentionality; on the other hand, the same observation suggests that the *what, when, and whether* segregation for action most likely exists beyond the concept of intentionality.

However, it cannot be denied that the presence of an intentional stance during the paradigms meta-analyzed here induced stronger activity in these regions in a component specific manner. This suggests, on the one hand, that intentionality is expressed in these *www* action regions at some microscopical level, perhaps thanks to the boosting effect of some ascending modulatory attentional pathways for tasks in which subjects have to decide how and whether to act by themselves, rather than in reaction to a conditional stimulus. Another possibility is that the relative weights of the connections between the regions involved in the different aspects of intentionality change during intentional action, something that needs to be explored with effective connectivity techniques and that goes beyond the potentials of meta-analyses based on data generated by univariate analyses.

## Conclusion and future directions

After this meta-analytic review, it is clear that further studies are needed to assess the functional anatomical foundations of the *www-model* of intentionality, in order to overcome the methodological limitations of previous attempts. In particular, we are much in need of novel fMRI experiments in which the sub-components of intentionality, postulated by the above mentioned neurocognitive models, are assessed with an uniform procedure in the same group of subjects. The advantages of a uniform procedure are obvious: significant differences between the intentional tasks in the different conditions should not be confounded by factors (e.g., different populations; different tasks; different scanning protocols) that may hamper the possibility of firm assignations of specific functional anatomical patterns to subcomponents of intentionality. Another unexplored issue is the characterization of intentional actions in conditions in which content, timing and the very decision on whether to act or not are explicitly and jointly manipulated. Furthermore, it remains to be discovered how general-purpose action brain regions become more active when intentionality is operating. This will clearly require analytical approaches that go beyond univariate analyses of the fMRI data. Clearly, one such ambitious model should have some predictive value for pathologies in which a disorder of intentionality and its specific components is expected. Brass and Haggard (2008) speculated about candidate pathologies that may entail a specific disorder of intentionality (e.g., the *when* component in Parkinson's disease). These specific associations also remain to be demonstrated convincingly. However, in spite of all these unsolved issues, we believe that the available literature contains sufficient evidence to think that the *www-model* of intentionality might be a useful starting framework for future investigations.

## Author contributions

LZ, SS, and EP reviewed the data for the meta-analyses, performed the analyses, and drafted the manuscript.

### Conflict of interest statement

The authors declare that the research was conducted in the absence of any commercial or financial relationships that could be construed as a potential conflict of interest.

## Footnotes

^1^In their words, the model comprises “a component related to the decision about which action to execute (what component), a component that is related to the decision about when to execute an action (when component), and finally the decision about whether to execute an action or not (whether component).”

^2^Because second level fMRI analyses imply the use of contrast images generated, at the very least, by comparison with an implicit baseline, comparisons of experimental conditions end up being interaction effects.

^3^These were the *what, when* and *whether* clusters defined on the BrainMap.org database using as seeds the centroids of the clusters of the hierarchical clustering analysis,

^4^The BrainMap.org database, as large as it is, has contents that are dependent on the input of a distributed set of users. At the time of writing, it contained data from 3066 papers with 118308 regional effects and 15155 statistical comparisons.

## Abbreviations

AAL: Automatic Anatomical Labeling
ALE: Activation likelihood estimation
CSTC: Cortico-striato-thalamo-cortical
EEG: Electroencephalography
fMRI: Functional magnetic resonance imaging
FDR: False Discovery Rate
HC: Hierarchical clustering
MNI: Montreal Neurological Institute
OCD: Obsessive-Compulsive Disorder
OCTD: Obsessive-Compulsive Tic Disorder
PD: Parkinson's Disease
PET: Positron emission tomography
Pre-SMA: Pre Supplementary motor area
WWW MODEL: What, When and Whether model
SMA: Supplementary motor area.
