# Supplementary material for: The What, the When, and the Whether of Intentional Action in the Brain: A Meta-Analytical Review
Source: Front Hum Neurosci. 2017 May 17;11:238. doi: 10.3389/fnhum.2017.00238 (PMC5434171; doi:10.3389/fnhum.2017.00238)
Supplement: Supplementary file 1 [file Table1.DOCX]

**Table s1. PET/fMRI studies included in the hierarchical clustering meta-analysis.**

| # | **First Author** | **Year** | **Component investigated** | **Technique** |
| --- | --- | --- | --- | --- |
| 1 | Deiber M P | 1991 | What | PET |
| 2 | Frith C D | 1991 | What | PET |
| 3 | Hyder F | 1997 | What | fMRI |
| 4 | Lau H C | 2004b | What | fMRI |
| 5 | Van Eimeren T | 2006 | What | fMRI |
| 6 | Mueller V A | 2007 | What | fMRI |
| 7 | Hoffstaedter F | 2013 | What & When | fMRI |
| 8 | Krieghoff V | 2009 | What & When | fMRI |
| 9 | Jahanshahi M | 1995 | When | PET |
| 10 | Jenkins I H | 2000 | When | PET |
| 11 | Lau H C | 2004a | When | fMRI |
| 12 | Brass M | 2007 | Whether | fMRI |
| 13 | Kuhn S | 2009 | Whether | fMRI |
| 14 | Lynn M T | 2014 | Whether | fMRI |
| 15 | Schel M A | 2014 | Whether | fMRI |
