# Supplementary material for: The What, the When, and the Whether of Intentional Action in the Brain: A Meta-Analytical Review
Source: Front Hum Neurosci. 2017 May 17;11:238. doi: 10.3389/fnhum.2017.00238 (PMC5434171; doi:10.3389/fnhum.2017.00238)
Supplement: Supplementary file 2 [file Table2.docx]

**Table s2a. Studies that contributed to the local maxima inside the median wall “what specific” cluster**

| **Cluster** | **Author, year** | **Journal** | **PMID** | **Peaks** | **Paradigm Class** |
| --- | --- | --- | --- | --- | --- |
| 1 | Grafton S T, 2001 | Journal of Cognitive Neuroscience | 11244547 | 1 | Action Motor Learning |
| 2 | Stephan K M, 2002 | NeuroImage | 11798270 | 1 | Finger Tapping/Button Press |
| 3 | Decety J, 1994 | Nature | 7935791 | 2 | Imagined Movement |
| 4 | O'Driscoll G A, 1995 | Proceedings of the National Academy of Sciences | 7846080 | 1 | Anti-Saccades |
| 5 | Xiong J, 2000 | NeuroImage | 10944415 | 1 | Word Generation (Overt) |
| 6 | Tan L H, 2001 | Neuroreport | 11201097 | 2 | Reading (Overt) |
| 7 | Tan L H, 2001 | Neuroreport | 11201097 | 2 | Reading (Overt) |
| 8 | Rubia K, 2001 | NeuroImage | 11162266 | 2 | Go/No-Go |
| 9 | Paus T, 1993 | Journal of Neurophysiology | 8410148 | 1 | Anti-Saccades |
| 10 | Seitz R J, 2000 | Experimental Brain Research | 10933212 | 1 | Finger Tapping/Button Press |
| 11 | Kawashima R, 1996 | Brain Research | 8864300 | 1 | Go/No-Go (multiple effectors) |
| 12 | Murtha S, 1999 | Journal of Cognitive Neuroscience | 10471848 | 1 | Discrimination |
| 13 | Astafiev S V, 2003 | Journal of Neuroscience | 12805308 | 1 | Pointing |
| 14 | Garavan H, 1999 | Proceedings of the National Academy of Sciences | 10393989 | 1 | Go/No-Go |
| 15 | Kiehl K A, 2000 | Psychophysiology | 10731771 | 1 | Go/No-Go |
| 16 | Kiehl K A, 2000 | Psychophysiology | 10731771 | 1 | Go/No-Go |
| 17 | Kiehl K A, 2000 | Psychophysiology | 10731771 | 1 | Go/No-Go |
| 18 | Ford K A, 2005 | Journal of Neurophysiology | 15728770 | 1 | Anti-Saccades |
| 19 | MacDonald III A W, 2003 | Journal of Abnormal Psychology | 14674880 | 1 | N-back |
| 20 | Basho S, 2007 | Neuropsychologia | 17292926 | 1 | Word Generation (Covert), Word Generation (Overt) |
| 21 | Bohland J W, 2006 | NeuroImage | 16730195 | 1 | Go/No-Go |
| 22 | Kirsch P, 2003 | NeuroImage | 14568478 | 1 | Reward |
| 23 | Kirsch P, 2003 | NeuroImage | 14568478 | 1 | Reward |
| 24 | Martin R E, 2004 | Journal of Neurophysiology | 15163677 | 1 | Chewing/Swallowing |
| 25 | Goldin P R, 2008 | Biological Psychiatry | 17888411 | 1 | Facial expression inhibition |
| 26 | Brown M R G, 2006 | NeuroImage | 16949303 | 1 | Saccades |
| 27 | Brown M R G, 2006 | NeuroImage | 16949303 | 1 | Anti-Saccades |
| 28 | Brown M R G, 2006 | NeuroImage | 16949303 | 1 | Anti-Saccades |
| 29 | Brown M R G, 2007 | Journal of Neurophysiology | 17596416 | 1 | Anti-Saccades |
| 30 | Rektorova I, 2007 | Movement Disorders | 17683056 | 1 | Reading (Overt) |
| 31 | Rektorova I, 2007 | Movement Disorders | 17683056 | 1 | Reading (Overt) |
| 32 | Rektorova I, 2008 | Movement Disorders | 17683056 | 1 | Reading (Overt) |
| 33 | Martin R, 2007 | Experimental Brain Research | 16896984 | 1 | Chewing/Swallowing |
| 34 | Tourville J A, 2008 | NeuroImage | 18035557 | 1 | Reading (Overt) |
| 35 | Lee T W, 2006 | Social Cognitive and Affective Neuroscience | 17356686 | 1 | Imitation |
| 36 | Lee T W, 2006 | Social Cognitive and Affective Neuroscience | 17356686 | 1 | Imitation |
| 37 | Chikazoe J, 2009 | Journal of Neuroscience | 20016103 | 2 | Go/No-Go |
| 38 | Hester R L, 2009 | Neuropsychopharmacology | 19553917 | 1 | Go/No-Go, n-back, Stroop-Color Word |
| 39 | Simoes-Franklin C, 2010 | Human Brain Mapping | 19718655 | 1 | Go/No-Go |
| 40 | Simoes-Franklin C, 2010 | Human Brain Mapping | 19718655 | 1 | Go/No-Go |
| 41 | De Nil L F, 2008 | Brain and Language | 18822455 | 1 | Repetition (Overt) |
| 42 | Singh M K, 2010 | Journal of Child and Adolescent Psychopharmacology | 20166792 | 1 | Go/No-Go |
| 43 | Townsend J D, 2012 | Bipolar Disorders | 22631623 | 1 | Go/No-Go |
| 44 | Zandbelt B B, 2011 | Biological Psychiatry | 21903198 | 2 | Go/No-Go |
| 45 | Zandbelt B B, 2011 | Biological Psychiatry | 21903198 | 2 | Go/No-Go |
| 46 | Zandbelt B B, 2011 | Biological Psychiatry | 21903198 | 1 | Go/No-Go |
| 47 | Rapp A, 2004 | Cognitive Brain Research | 15268917 | 1 | Discrimination |
| 48 | Shah C, 2011 | Human Brain Mapping | 22162145 | 1 | Reading (Covert) |
| 49 | Martinsen S, 2014 | PLoS ONE | 25275449 | 1 | Stroop-Color Word, Finger Tapping/Button Press |
| 50 | Brendel B, 2011 | Motor Control | 21339513 | 1 | Reading (Covert) |
| 51 | Heim S, 2009 | Human Brain Mapping | 18344173 | 1 | Naming (Overt) |
| 52 | Shafritz K M, 2015 | Progress In Neuro-Psychopharmacology & Biological Psychiatry | 25765593 | 1 | Go/No-Go |
| 53 | Zarate J M, 2010 | Neuropsychologia | 19896958 | 2 | Discrimination |
| 54 | Zarate J M, 2010 | Neuropsychologia | 19896958 | 2 | Discrimination |
| 55 | Zarate J M, 2010 | Neuropsychologia | 19896958 | 2 | Discrimination |
| 56 | Lacourse M G, 2005 | NeuroImage | 16046149 | 1 | Imagined Movement |
|  |  |  |  |  |  |
|  |  |  |  |  |  |

**Table s2b. Studies that contributed to the local maxima inside the median wall “when specific” cluster**

| **Cluster** | **Author, year** | **Journal** | **PMID** | **Peaks** | **Paradigm Class** |
| --- | --- | --- | --- | --- | --- |
| 1 | Sadato N, 1998 | Brain | 9679774 | 1 | Tactile Monitor/Discrimination |
| 2 | Gerardin E, 2000: | Cerebral Cortex | 11053230 | 2 | Finger Tapping/Button Press |
| 3 | Gitelman D R, 2002: | NeuroImage | 11906237 | 1 | Saccades |
| 4 | Seitz R J, 2000: | Experimental Brain Research | 10933212 | 1 | Finger Tapping/Button Press |
| 5 | Umetsu A, 2002: | NeuroImage | 12482091 | 2 | Flexion/Extension |
| 6 | Ingham R J, 2000: | Brain and Language | 11049665 | 1 | Reading (Overt) |
| 7 | Ingham R J, 2000: | Brain and Language | 11049665 | 1 | Reading (Overt) |
| 8 | Lerner A, 2004: | NeuroImage | 15006657 |  | Writing - Rest |
| 9 | Meister I, 2005: | Human Brain Mapping | 15852385 | 2 | Sequence Recall/Learning, Music Comprehension |
| 10 | Meister I, 2005: | Human Brain Mapping | 15852385 | 1 | Sequence Recall/Learning, Music Comprehension |
| 11 | Berman R A, 1999: | Human Brain Mapping | 10619415 | 4 | Visual Pursuit/Tracking, Saccades |
| 12 | Law I, 1998: | Brain | 9827777 | 1 | Saccades |
| 13 | Gagnon D, 2002: | Brain | 11834598 | 1 | Saccades |
| 14 | Gagnon D, 2002: | Brain | 11834598 | 1 | Saccades |
| 15 | Sweeney J A, 1996: | Journal of Neurophysiology | 8822570 | 2 | Saccades |
| 16 | Thulborn K R, 2000: | American Journal of Neuroradiology | 10730646 | 2 | Saccades |
| 17 | Soros P, 2006: | NeuroImage | 16631384 | 1 | Recitation/Repetition (Overt) |
| 18 | van der Graaf F H C E, 2004: | Cognitive Brain Research | 15183385 | 1 | Sequence Recall/Learning |
| 19 | Dapretto M, 2006: | Nature Neuroscience | 16327784 | 1 | Monitor/Discrimination, Flexion/Extension |
| 20 | Wong S W, 2007: | NeuroImage | 17291781 | 1 | Isometric Force |
| 21 | Bohland J W, 2006: | NeuroImage | 16730195 | 1 | Go/No-Go, Recitation/Repetition (Overt) |
| 22 | Blok B F M, 1997: | Journal of Comparative Neurology | 9414011 | 1 | Flexion/Extension |
| 23 | Lissek S, 2007: | NeuroImage | 7110327 | 2 | Finger Tapping/Button Press |
| 24 | Lissek S, 2007: | NeuroImage | 7110327 | 2 | Finger Tapping/Button Press |
| 25 | Lissek S, 2007: | NeuroImage | 7110327 | 2 | Finger Tapping/Button Press |
| 26 | Cross E S, 2007: | Journal of Cognitive Neuroscience | 17958488 | 3 | Go/No-Go, Sequence Recall/Learning |
| 27 | Johnson S H, 2002: | NeuroImage | 12498743 | 1 | Imagined Movement |
| 28 | Stephan K M, 1995: | Journal of Neurophysiology | 7714579 | 3 | Imagined Movement |
| 29 | Gerardin E, 2003: | Cerebral Cortex | 12507947 | 1 | Flexion/Extension |
| 30 | Gerardin E, 2003: | Cerebral Cortex | 12507948 | 2 | Flexion/Extension |
| 31 | Gerardin E, 2003: | Cerebral Cortex | 12507949 | 1 | Saccades |
| 32 | Rotte M, 2002: | Stereotactic and Functional Neurosurgery | 12381881 | 1 | Flexion/Extension |
| 33 | Lowell S Y, 2008: | NeuroImage | 18515150 | 2 | Imagined Movement |
| 34 | Siebner H R, 2002: | Journal of Neuroscience | 11923446 | 1 | Writing |
| 35 | Fraser C, 2002: | Neuron | 12062028 | 1 | Chewing/Swallowing |
| 36 | Fraser C, 2002: | Neuron | 12062029 | 1 | Chewing/Swallowing |
| 37 | Guillot A, 2009: | Human Brain Mapping | 18819106 | 4 | Finger Tapping/Button Press |
| 38 | Guillot A, 2009: | Human Brain Mapping | 18819107 | 4 | Imagined Movement |
| 39 | Guillot A, 2009: | Human Brain Mapping | 18819108 | 3 | Imagined Movement |
| 40 | Martin R, 2007: | Experimental Brain Research | 16896984 | 1 | Chewing/Swallowing |
| 41 | Lee T W, 2006: | SCAN | 17356686 | 2 | Face Monitor/Discrimination |
| 42 | Seseke S, 2008: | NeuroImage | 18721889 | 1 | Flexion/Extension, Micturition |
| 43 | Brown S, 2006: | Cerebral Cortex | 16221923 | 2 | Flexion/Extension |
| 44 | De Boissezon X, 2009: | European Journal of Physical and Rehabilitation Medicine | 20032914 | 1 | Naming (Overt) |
| 45 | Yu H, 2007: | NeuroImage | 17223579 | 2 | Finger Tapping/Button Press |
| 46 | Yu H, 2007: | NeuroImage | 17223579 | 2 | Finger Tapping/Button Press |
| 47 | Yu H, 2007: | NeuroImage | 17223579 | 2 | Finger Tapping/Button Press |
| 48 | Chikazoe J, 2009: | Cerebral Cortex | 18445602 | 2 | Go/No-Go |
| 49 | Chikazoe J, 2009: | Cerebral Cortex | 18445602 | 2 | Go/No-Go |
| 50 | Lerner A, 2007: | Neurology | 17548547 | 1 | Rest |
| 51 | Lerner A, 2007: | Neurology | 17548547 | 1 | Rest |
| 52 | Simoes-Franklin C, 2010: | Human Brain Mapping | 19718655 | 1 | Go/No-Go |
| 53 | Grabski K, 2011: | Human Brain Mapping | 21826760 | 2 | Flexion/Extension |
| 54 | Ogura E, 2012: | Dysphagia | 22076444 | 2 | Flexion/Extension |
| 55 | Wong D, 2011: | Brain Research | 21295015 | 3 | Flexion/Extension |
| 56 | Wong D, 2011: | Brain Research | 21295015 | 2 | Flexion/Extension |
| 57 | Wong D, 2011: | Brain Research | 21295016 | 1 | Flexion/Extension |
| 58 | Ingham R J, 2012: | Brain and Language | 22564749 | 1 | Word generation (Over) |
| 59 | Zandbelt B B, 2011: | Biological Psychiatry | 21903198 | 3 | Go/No-Go |
| 60 | Guo X, 2013: | Brain and Cognition | 23673251 | 1 | Imagined Objects/Scenes |
| 61 | Blickenstorfer A, 2009: | Human Brain Mapping | 18344193 | 1 | Flexion/Extension |
| 62 | Baerentsen K B, 2010: | Cognitive Processing | 19876663 | 3 | Meditation |
| 63 | Parkinson A L, 2012: | NeuroImage | 22406500 | 1 | Pitch Monitor/Discrimination |
| 64 | Peran P, 2009: | Cortex | 19368905 | 1 | Naming (Overt) |
| 65 | Zarate J M, 2010: | Neuropsychologia | 19896958 | 2 | Pitch Monitor/Discrimination |
| 66 | Boecker H, 1998: | Journal of Neurophysiology | 9463462 | 1 | Finger Tapping/Button Press |
| 67 | Kuhtz-Buschbeck J P, 2003: | European Journal of Neuroscience | 14686911 | 1 | Imagined Movement |
| 68 | Sadato N, 1997: | Journal of Neuroscience | 9391021 | 1 | Finger Tapping/Button Press |
| 69 | Dettmers C, 1995: | Journal of Neurophysiology | 7472384 | 4 | Finger Tapping/Button Press |
| 70 | Lacourse M G, 2005: | NeuroImage | 16046149 | 1 | Finger Tapping/Button Press |
| 71 | Lacourse M G, 2005: | NeuroImage | 16046150 | 1 | Finger Tapping/Button Press |
| 72 | Lutz K, 2000: | Neuroreport | 10817611 | 1 | Finger Tapping/Button Press |
| 73 | Lutz K, 2000: | Neuroreport | 10817612 | 2 | Finger Tapping/Button Press |
|  |  |  |  |  |  |
|  |  |  |  |  |  |

| **Table s2c. Studies that contributed to the local maxima inside the median wall “whether specific” cluster** |
| --- |

|  | | | | | |
| --- | --- | --- | --- | --- | --- |
| **Cluster** | **Author, year** | **Jornal** | **PMID** | **Peaks** | **Paradigm Class** |
| 1 | Decety J, 1994: | Nature | 7935791 | 1 | Imagined Movement |
| 2 | Decety J, 1994: | Nature | 7935791 | 4 | Imagined Movement |
| 3 | Xiong J, 2000: | NeuroImage | 10944415 | 1 | Word Generation (Overt) |
| 4 | Bookheimer S Y, 1995: | Human Brain Mapping | -- | 1 | Naming (Overt) |
| 5 | Braun A R, 1997: | Brain | 9183248 | 2 | Word Generation (Overt) |
| 6 | Braun A R, 1997: | Brain | 9183248 | 1 | Word Generation (Overt) |
| 7 | Paus T, 1993: | Journal of Neurophysiology | 8410148 | 1 | Discrimination |
| 8 | Kawashima R, 1996: | Brain Research | 8864300 | 1 | Go/No-Go (multiple effectors) |
| 9 | Price C J, 1996: | Brain | 8673502 | 1 | Repetition (Overt) |
| 10 | Kelly A M, 2004: | European Journal of Neuroscience | 15182319 | 1 | Go/No-Go (letters) |
| 11 | Carter C S, 2001: | American Journal of Psychiatry | 11532726 | 1 | Go/No-Go |
| 12 | Parris B A, 2007: | Journal of Cognitive Neuroscience | 17214559 | 1 | Task Switching |
| 13 | Parris B A, 2007: | Journal of Cognitive Neuroscience | 17214559 | 1 | Task Switching |
| 14 | Herholz K, 2002: | NeuroImage | 12482085 | 1 | Rest |
| 15 | Mensebach C, 2009: | Psychiatry Research | 19176280 | 1 | Word Generation (Overt) |
| 16 | Caffarra P, 2008: | Open Neuroimaging Journal | 19018314 | 1 | Rest |
| 17 | Chikazoe J, 2009: | Journal of Cognitive Neuroscience | 17214564 | 1 | Go/No-Go (shapes) |
| 18 | Strakowski S M, 2008: | Early Intervention in Psychiatry | 19190727 | 1 | Go/No-Go (letters) |
| 19 | Strakowski S M, 2008: | Early Intervention in Psychiatry | 19190727 | 1 | Go/No-Go (letters) |
| 20 | Zandbelt B B, 2011: | Biological Psychiatry | 21903198 | 1 | Go/No-Go (shapes) |
| 21 | Zandbelt B B, 2011: | Biological Psychiatry | 21903198 | 2 | Go/No-Go (shapes) |
| 22 | Zandbelt B B, 2011: | Biological Psychiatry | 21903198 | 1 | Go/No-Go (shapes) |
| 23 | Bruhl A B, 2011: | Psychopharmacology | 21359508 | 1 | Passive Viewing, Affective Pictures |
